# Supplementary material for: Limited long-term cooling effects of Pangaean flood basalt weathering
Source: Nat Commun. 2025 May 23;16:4813. doi: 10.1038/s41467-025-59480-0 (PMC12102204; doi:10.1038/s41467-025-59480-0)
Supplement: Supplementary file 1 — Supplementary Information [file 41467_2025_59480_MOESM1_ESM.pdf]

Supplementary Materials for "Limited long-term cooling effects of Pangaean flood basalt weathering" by Jack Longman et al.

Supplementary Text

To include LIP-extents within *SCION* as weatherable features, we use the digital compilation of ref.[1]. In their contribution, ref.[1] estimate the full original extent of LIP emplacement, rather than just restoring the present-day extents. Using these LIP polygons, we reconstruct them to their time of eruption using the palaeogeographic model of ref.[2] and *pyGPlates* and *GPlates* (www.gplates.org), allowing us to define their position from their eruption time to present-day. At the *SCION*-model times we then extract the spatial position of the LIPs and rasterise the LIP polygons at the grid-cell resolution of the *SCION* model (4.5° • 7.5°) (Fig. 3, Figure S2). Where the LIP polygon boundaries intersect our grid-cells we calculate the fractional area that is included within that cell. At each *SCION*-model time we also calculate the expected decay of the LIP to determine the fractional content that remains, using a LIP half-life of 29 Ma as established by ref.[1]. Supplementary Figure 3 shows the evolution of the Siberian Traps from the earliest *SCION*-model time post emplacement (245 Ma), to present-day.

Once our grid-cells at each model-step are determined, we add these as a layer into the *SCION* model via addition of "LIPSTACK" as a forcing, and each LIP as a global variable. The *SCION* code loads each LIP as a terrane of known location and with a value related to the proportion of each grid cell covered with LIP material from each LIP at each time point. This value is then multiplied by the LIP multiplier (in this case 7, a value used in previous work[3,4] and based on experimental estimates[5]), and erosion is calculated by multiplying each grid square's erosion by this value.

Supplementary Figures

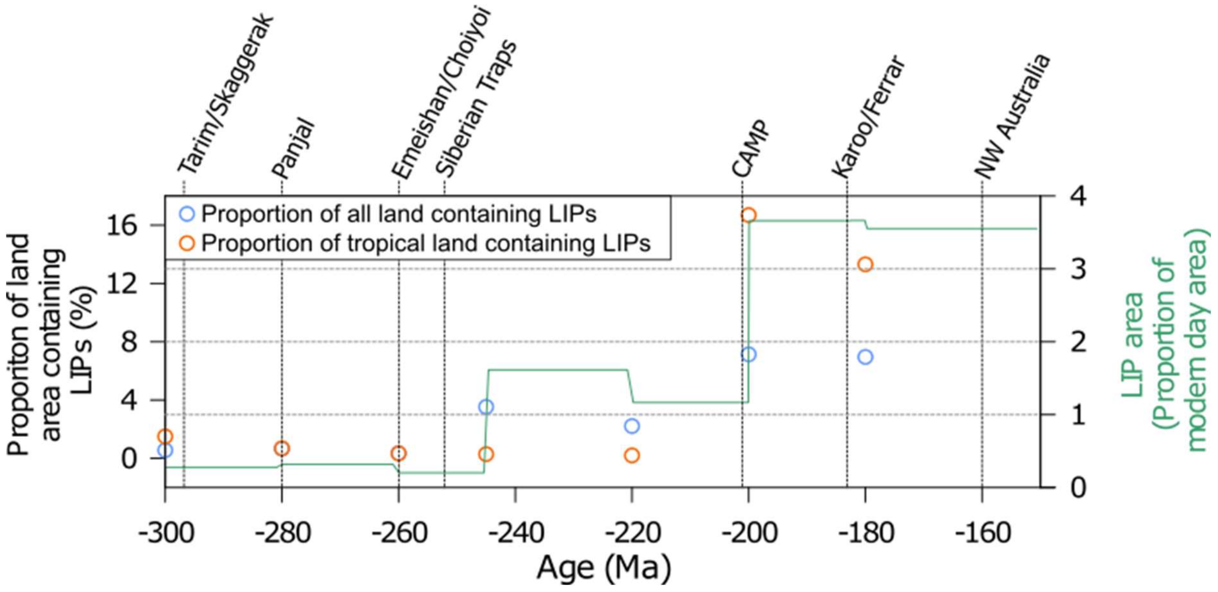

Fig. S1.

The percentage of terrestrial grid squares in each GCM run to contain some LIP material (blue circles) and the percentage of these squares in the tropics which contain LIP material (orange circles). Plotted alongside relative LIP area versus present (green)**.**

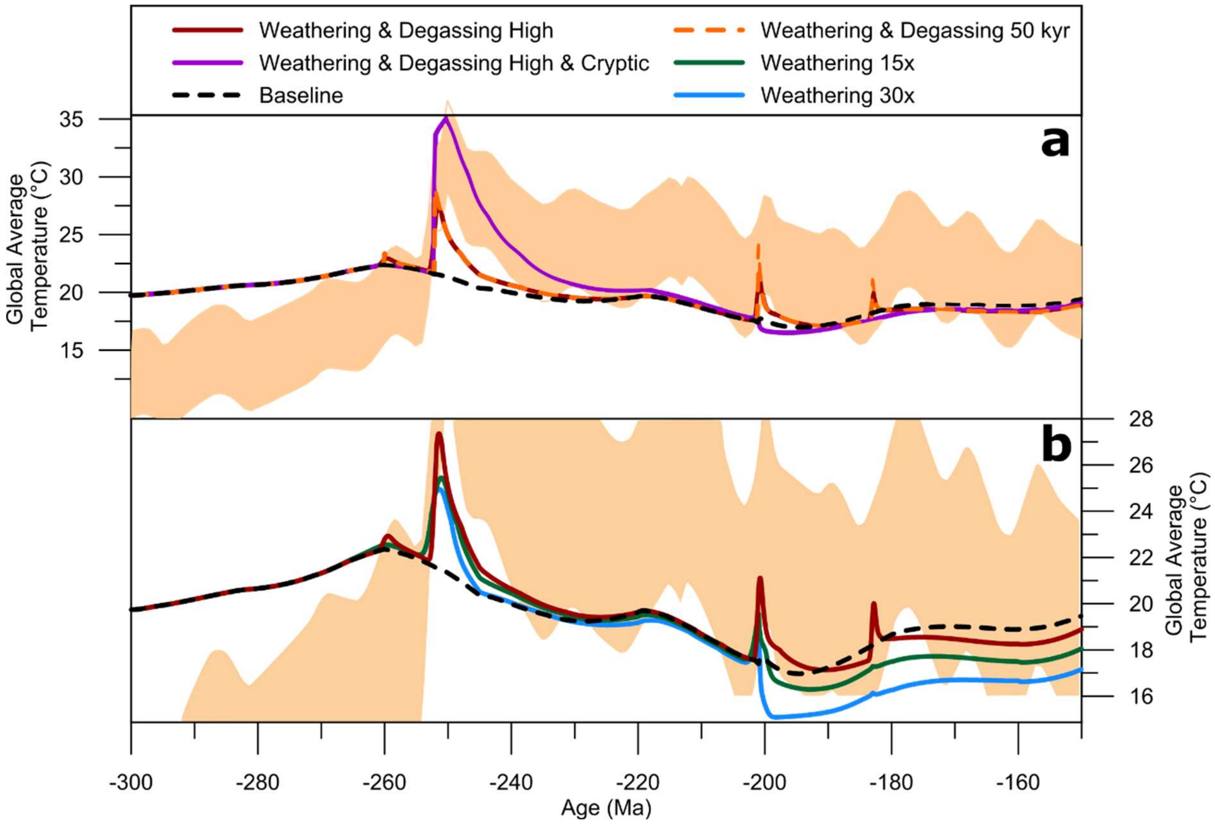

Fig. S2.

Comparison of model outputs of temperature for a range of degassing (a) and higher weathering enhancement (b) scenarios. See Table S1 for further information, and the Main Text for a discussion of the results.

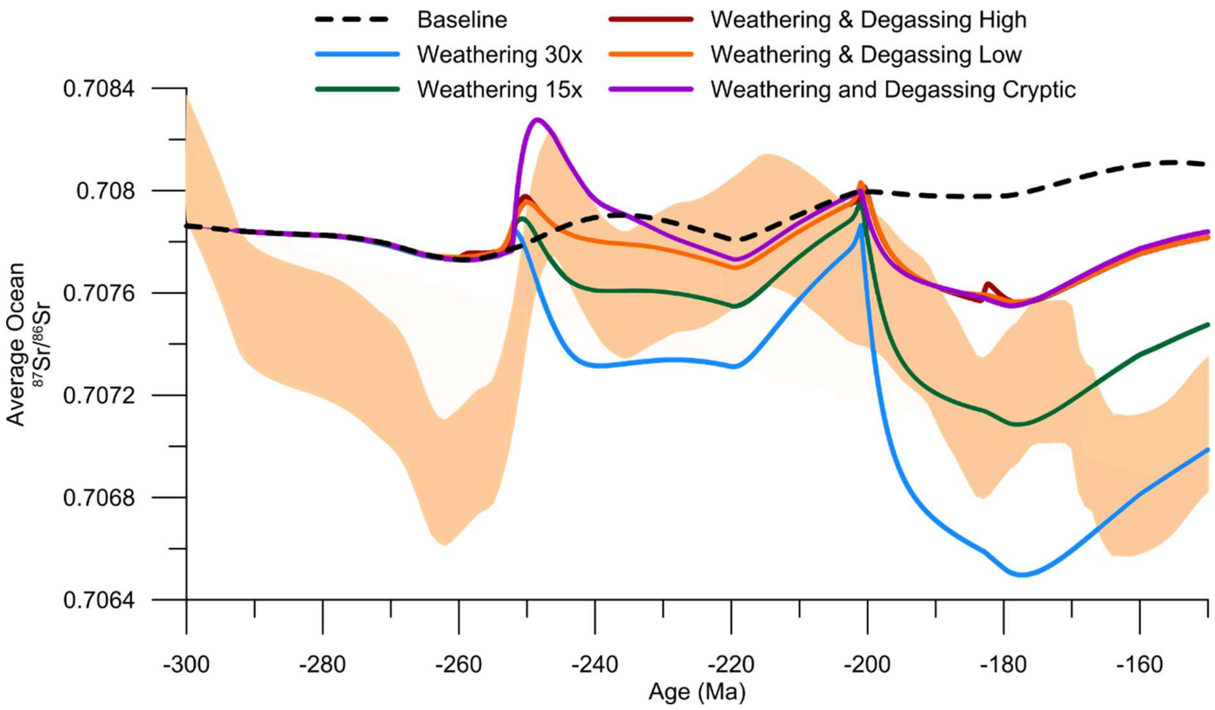

Fig. S3.

Comparison of model outputs of average ocean $^{87}Sr/^{86}Sr$ for a range of degassing (a) and higher weathering enhancement (b) scenarios. See Table S1 for further information, and the Main Text for a discussion of the results.

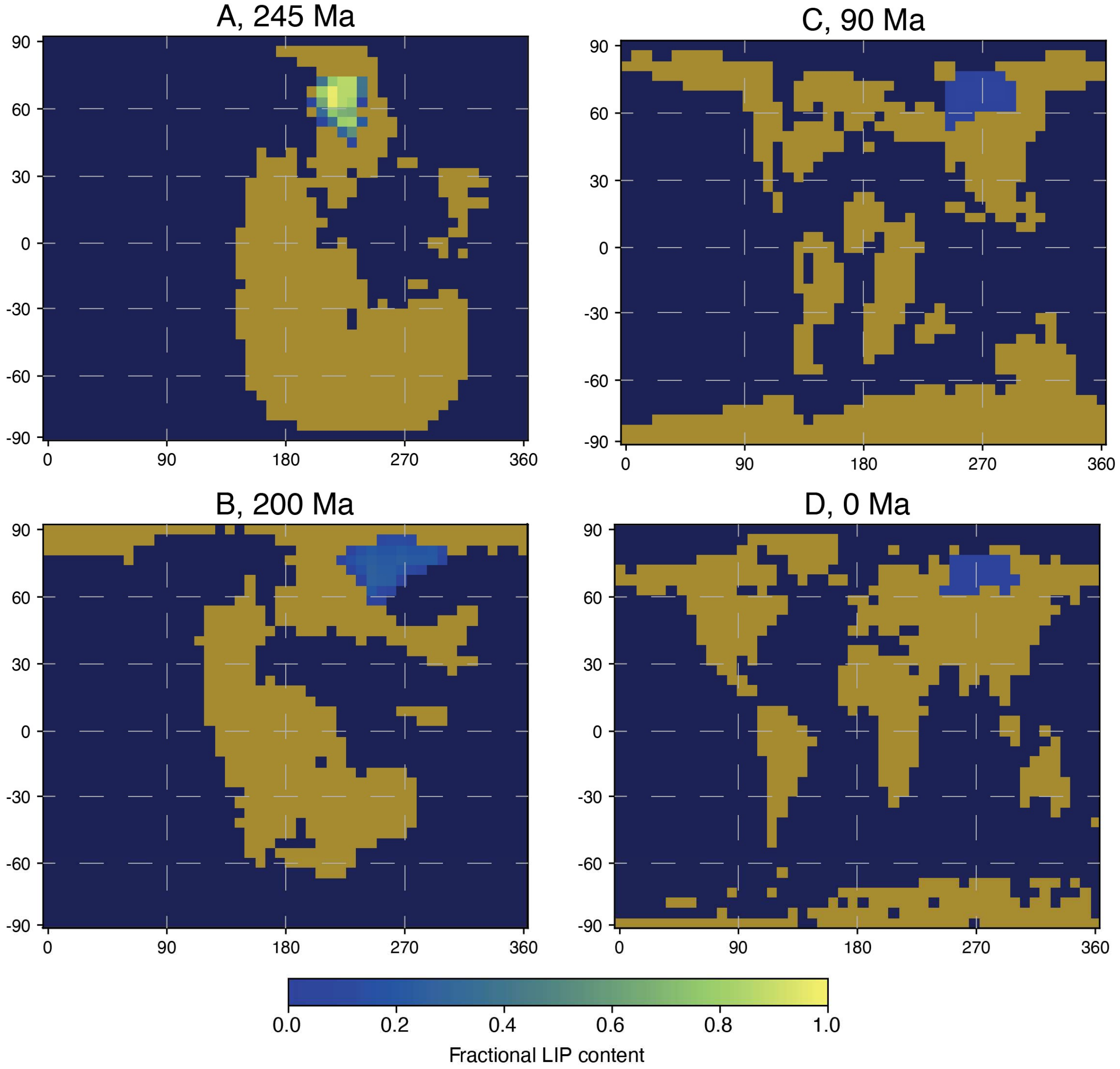

Fig. S4.

Evolution of the Siberian Traps from the earliest SCION-model time post emplacement (245 Ma), to present-day. In each panel, the proportion of land covered by Siberian Traps material is indicated by coloured shading. This proportion value is used to calculate the enhancement of the weathering of each grid cell. Panels relate to the location and evolution of the LIP in the model at 245 Ma (a), 200 ma (b), 90 Ma (c) and present (d).

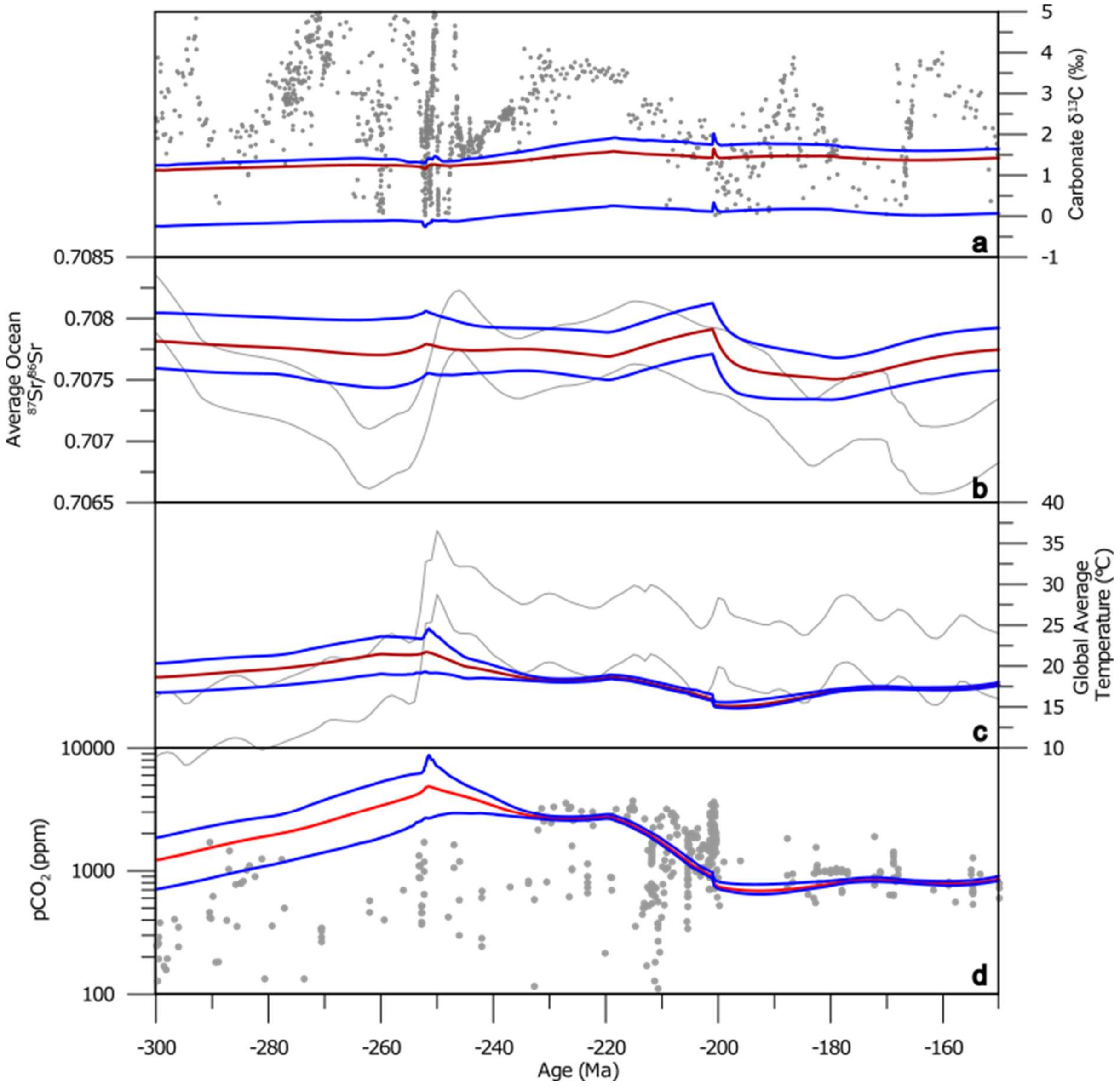

Fig. S5.

Output of sensitivity test, whereby 1000 simulations of the scenario Weathering & Degassing High (Table S2) model were run, with Monte Carlo style variation of the granite area, background degassing, and carbon isotope composition of each of the reservoirs (See ref.[6] and the Methods for more information). In each panel the red line is the mean of the ensemble of simulations and the blue lines denote ±1 SD of the mean.

Table S1.

Model scenarios tested in this work. Unless stated, the weathering parameter is considers LIP material to be seven times more weatherable than other rock. The degassing parameter 'low' refers to the lower bound estimate in Table S2, and 'high' the highest bound. The degassing parameter 'High & Cryptic' considers the 'high' degassing scenario, but with the addition of cryptic degassing in line with ref.[7]. The scenario with 'set Gaussians' assumes the 'high' degassing rate, but that all degassing occurred over only 50 kyr.

Scenario	Code	Weathering Parameter	Degassing Parameter
Baseline	B	None	None
Weathering Only	W	Included	None
Degassing Low Only	DL	None	Low
Degassing High Only	DH	None	High
Degassing High & Cryptic Only	DC	None	High & Cryptic
Weathering & Degassing Low	W & DL	Included	Low
Weathering & Degassing High	W & DH	Included	High
Weathering & Degassing High & Cryptic	W & DC	Included	High & Cryptic
Weathering & Degassing 50 kyr	W & D 50	Included	Set Gaussians 50kyr
Weathering & Degassing x30	W & D x30	Included, 30-fold increase	High

Table S2.

Ranges of carbon emission data used to force the model. For each Large Igneous Province the timing, the volume, amount of carbon and degassing rate are presented. For each LIP, a 'low' and 'high' scenario is presented, see Methods from further information. All information is from previously published studies, cited here.

Name of LIP	Name in *SCION* model	Time In Model (Ma)	Volume (Mkm^3)		Duration (Myr)		Carbon Emission (mol C)		Degassing Rate (mol C/yr)	
			Low	High	Low	High	Low	High	Low	High
NW Australian Margin /	nwaus	165	0.32[a]	0.39[a]	10[a]	10[a]	3E+16	3E+16	2.6E+09	3.2E+09

Gascoyne Margin										
Karoo	karoo	183	1 (ref.[8])	2.5 (ref.[9])	0.47 (ref.[10])	6.5 (ref.[11])	3E+16	2E+17	4.7E+09	3.6E+12 [b]
Ferrar	ferrar	183	0.15 (ref.[12])	0.57 (ref.[13])	0.35 (ref.[14])	0.5 (ref.[15])	1E+16	5E+16	2.5E+10	1.3E+11
Central Atlantic Magmatic Province	camp	201	2 (ref.[16])	4 (ref.[17,18])	0.6 (ref.[19])	1.5 (ref.[20])	2E+17	3E+17	1.1E+11	8.3E+12 [c]
Siberian Traps	siberian	252	1.7 (ref.[21])	11 (ref.[22])	0.8 (ref.[23])	2 (ref.[24])	6E+17	1E+18	2.9E+11	1.7E+13 [d]
Emeishan	emeishan	260	0.35 (ref.[25])	8.9 (ref. [26])	1 (ref.[25])	7 (ref.[27])	3E+16	7E+17	3.6E+09	1.7E+12 [e]
Panjal - Qiangtang	panjal	280	0.1 (ref.[28])	0.75 (ref.[29])	5 (ref.[30])	30 (ref.[31])	8E+15	6E+16	2.8E+08	1.3E+10
Tarim	tarim	300	0.15 (ref.[32])	0.3 (ref.[33])	5 (ref.[34])	40 (ref.[15])	1E+16	3E+16	3.1E+08	5.0E+09

[a] Only a single estimate of volume[35] and duration[36] are available. To derive a ‘high’ and ‘low’ scenario, these values are derived from the volume estimate ± 10%.

[b] Rate calculated using estimate of ref.[37]

[c] Rate calculated using estimate of ref.[38].

[d] Rate calculated using estimate of ref.[39]

[e] Rate calculated using estimate of ref.[40]

References

1. Park, Y., Swanson-Hysell, N. L., Lisiecki, L. E. & Macdonald, F. A. Evaluating the Relationship Between the Area and Latitude of Large Igneous Provinces and Earth's Long-Term Climate State. *Large Igneous Provinces: A Driver of Global Environmental and Biotic Changes* 153–168 (2021) doi:10.1002/9781119507444.CH7.

2. Torsvik. Trond H. & Cocks, R. M. *Earth History and Palaeogeography*. (Cambridge University Press, Cambridge, 2016).

3. Lefebvre, V., Donnadieu, Y., Goddéris, Y., Fluteau, F. & Hubert-Théou, L. Was the Antarctic glaciation delayed by a high degassing rate during the early Cenozoic? *Earth Planet Sci Lett* **371–372**, 203–211 (2013).

4. Longman, J., Mills, B. J. W., Donnadieu, Y. & Goddéris, Y. Assessing Volcanic Controls on Miocene Climate Change. *Geophys Res Lett* **49**, (2022).

5. Dessert, C., Dupré, B., Gaillardet, J., François, L. M. & Allègre, C. J. Basalt weathering laws and the impact of basalt weathering on the global carbon cycle. *Chem Geol* **202**, 257–273 (2003).

6. Mills, B. J. W., Donnadieu, Y. & Goddéris, Y. Spatial continuous integration of Phanerozoic global biogeochemistry and climate. *Gondwana Research* (2021) doi:10.1016/j.gr.2021.02.011.

7. Black, B. A. *et al.* Cryptic degassing and protracted greenhouse climates after flood basalt events. *Nat Geosci* **17**, 1162–1168 (2024).

8. White, R. S. Mantle plume origin for the Karoo and Ventersdorp flood basalts, South Africa. *Journal of South African Geology* **100**, 271–282 (1997).

9. Storey, B. C. & Kyle, P. R. An active mantle mechanism for Gondwana breakup. *South African Journal of Geology* **100**, 283–290 (1997).

10. Svensen, H., Corfu, F., Polteau, S., Hammer, Ø. & Planke, S. Rapid magma emplacement in the Karoo Large Igneous Province. *Earth Planet Sci Lett* **325–326**, 1–9 (2012).

11. Greber, N. D. *et al.* New high precision U-Pb ages and Hf isotope data from the Karoo large igneous province; implications for pulsed magmatism and early Toarcian environmental perturbations. *Results in Geochemistry* **1**, 100005 (2020).

12. Hergt, J. M., Chappell, B. W., McCulloch, M. T., McDougall, I. & Chivas, A. R. Geochemical and Isotopic Constraints on the Origin of the Jurassic Dolerites of Tasmania. *Journal of Petrology* **30**, 841–883 (1989).

13. Fleming, T. H., Heimann, A., Foland, K. A. & Elliot, D. H. 40Ar/39Ar geochronology of Ferrar Dolerite sills from the Transantarctic Mountains, Antarctica: Implications for the age and origin of the Ferrar magmatic province. *Geol Soc Am Bull* **109**, 533–546 (1997).

14. Burgess, S. D., Bowring, S. A., Fleming, T. H. & Elliot, D. H. High-precision geochronology links the Ferrar large igneous province with early-Jurassic ocean anoxia and biotic crisis. *Earth Planet Sci Lett* **415**, 90–99 (2015).

15. Jiang, Q., Jourdan, F., Olierook, H. K. H. & Merle, R. E. An appraisal of the ages of Phanerozoic large igneous provinces. *Earth Sci Rev* **237**, 104314 (2023).

16. McHone, J. G. Volatile emissions from Central Atlantic Magmatic Province Basalts: Mass assumptions and environmental consequences. in 241–254 (2003). doi:10.1029/136GM013.

17. Marzoli, A. *et al.* The Central Atlantic Magmatic Province (CAMP): A Review. in 91–125 (2018). doi:10.1007/978-3-319-68009-5_4.

18. Holbrook, W. S. & Kelemen, P. B. Large igneous province on the US Atlantic margin and implications for magmatism during continental breakup. *Nature* **364**, 433–436 (1993).

19. Blackburn, T. J. *et al.* Zircon U-Pb Geochronology Links the End-Triassic Extinction with the Central Atlantic Magmatic Province. *Science (1979)* **340**, 941–945 (2013).

20. Nomade, S. *et al.* Chronology of the Central Atlantic Magmatic Province: Implications for the Central Atlantic rifting processes and the Triassic–Jurassic biotic crisis. *Palaeogeogr Palaeoclimatol Palaeoecol* **244**, 326–344 (2007).

21. Vasil'ev, Y. R., Zolotukhin, V. V., Feoktistov, G. D. & Prusskaya, S. N. Evaluation of the volumes and genesis of Permo-Triassic trap magmatism on the Siberian Platform. *Geologiya i geofizika* **41**, 1696–1705 (2000).

22. Saunders, A. D. Two LIPs and two Earth-system crises: The impact of the North Atlantic Igneous Province and the Siberian Traps on the Earth-surface carbon cycle. *Geological Magazine* vol. 153 201–222 Preprint at https://doi.org/10.1017/S0016756815000175 (2016).

23. Burgess, S. D. & Bowring, S. A. High-precision geochronology confirms voluminous magmatism before, during, and after Earth's most severe extinction. *Sci Adv* **1**, (2015).

24. Reichow, M. K. *et al.* The timing and extent of the eruption of the Siberian Traps large igneous province: Implications for the end-Permian environmental crisis. *Earth Planet Sci Lett* **277**, 9–20 (2009).

25. Shellnutt, J. G. The Emeishan large igneous province: A synthesis. *Geoscience Frontiers* **5**, 369–394 (2014).

26. Zhu, D., Luo, T.-Y., Gao, Z.-M. & Zhu, C.-M. Differentiation of the Emeishan Flood Basalts at the Base and throughout the Crust of Southwest China. *Int Geol Rev* **45**, 471–477 (2003).

27. Shellnutt, J. G., Pham, T. T., Denyszyn, S. W., Yeh, M.-W. & Tran, T.-A. Magmatic duration of the Emeishan large igneous province: Insight from northern Vietnam. *Geology* **48**, 457–461 (2020).

28. Ernst, R. E. & Buchan, K. L. Recognizing Mantle Plumes in the Geological Record. *Annu Rev Earth Planet Sci* **31**, 469–523 (2003).

29. Zhang, Y.-X. & Zhang, K.-J. Early Permian Qiangtang flood basalts, northern Tibet, China: A mantle plume that disintegrated northern Gondwana? *Gondwana Research* **44**, 96–108 (2017).

30. Dan, W. *et al.* Short duration of Early Permian Qiangtang-Panjal large igneous province: Implications for origin of the Neo-Tethys Ocean. *Earth Planet Sci Lett* **568**, 117054 (2021).

31. Liu, B. *et al.* Origin of Triassic mafic magmatism in the North Qiangtang terrane, central Tibetan Plateau: implications for the development of a continental back-arc basin. *J Geol Soc London* **177**, 826–842 (2020).

32. Xu, Y.-G., Wei, X., Luo, Z.-Y., Liu, H.-Q. & Cao, J. The Early Permian Tarim Large Igneous Province: Main characteristics and a plume incubation model. *Lithos* **204**, 20–35 (2014).

33. Tian, W. *et al.* The Tarim picrite–basalt–rhyolite suite, a Permian flood basalt from northwest China with contrasting rhyolites produced by fractional crystallization and anatexis. *Contributions to Mineralogy and Petrology* **160**, 407–425 (2010).

34. Chen, J. & Xu, Y. Permian Large Igneous Provinces and Their Paleoenvironmental Effects. in 417–434 (2021). doi:10.1002/9781119507444.ch18.

35. Rey, S. S., Planke, S., Symonds, P. A. & Faleide, J. I. Seismic volcanostratigraphy of the Gascoyne margin, Western Australia. *Journal of Volcanology and Geothermal Research* **172**, 112–131 (2008).

36. Symonds, P. A., Planke, S., Frey, O. & Skogseid, J. Volcanic evolution of the Western Australian continental margin and its implications for basin development. in *The Sedimentary Basins of Western Australia 2: Proceedings of the PESA Symposium,* (eds. Purcell, R. R. & Purcell, P. G.) 33–54 (Petroleum Exploration Society of Australia, Perth, 1998).

37. Heimdal, T. H., Goddéris, Y., Jones, M. T. & Svensen, H. H. Assessing the importance of thermogenic degassing from the Karoo Large Igneous Province (LIP) in driving Toarcian carbon cycle perturbations. *Nat Commun* **12**, 6221 (2021).

38. Capriolo, M. *et al.* Anthropogenic-scale CO2 degassing from the Central Atlantic Magmatic Province as a driver of the end-Triassic mass extinction. *Glob Planet Change* **209**, 103731 (2022).

39. Jiang, Q. *et al.* Volume and rate of volcanic CO_2 emissions governed the severity of past environmental crises. *Proceedings of the National Academy of Sciences* **119**, (2022).

40. Zhu, J. *et al.* Recycled carbon degassed from the Emeishan plume as the potential driver for the major end-Guadalupian carbon cycle perturbations. *Geoscience Frontiers* **12**, 101140 (2021).
